# Shear Wave Velocity: A New Quantitative Index to Estimate the Status of Thyroid in Diffuse Thyroid Disease

**DOI:** 10.1155/2015/626308

**Published:** 2015-06-03

**Authors:** Lin-Yao Du, Qiao Ji, Xiu-Juan Hou, Xiao-Lei Wang, Xian-Li Zhou

**Affiliations:** In-Patient Ultrasound Department, The Second Affiliated Hospital of Harbin Medical University, Harbin 150086, China

## Abstract

*Objective. *The purpose of the study was to assess the application value of VTQ in DTD.* Research Design and Methods.* Thirty healthy subjects and 74 DTD patients were involved. The thyroid stiffness, which was expressed by SWV, was measured by VTQ and compared between the patients and healthy people. The relationship between SWV and thyroid serological indexes was also analyzed.* Results*. The thyroid SWVs of DTD patients were higher than those of the healthy (2.56 ± 1.33 m/s versus 1.74 ± 0.16 m/s, *P* = 0.011). There was no significant difference between the thyroid SWVs in GD and HT patients (*P* = 0.168). The SWVs in patients with GD and HT were both higher than those of the healthy (*P* < 0.05). The area under the ROC curve was 0.938 for SWV to distinguish between DTD and healthy thyroid. With a cutoff value of 2.02 m/s, the sensitivity and specificity were 81.12% and 100.00%, respectively. Additionally, we found a positive liner correlation between thyroid SWV and TSH in DTD patients (*P* < 0.001).* Conclusion.* SWV is a good indicator of the thyroid tissue stiffness, which might be considered helpful in screening DTD. What is more, SWV might have a potential in assessing the thyroid function.

## 1. Introduction

**Figure 1:**
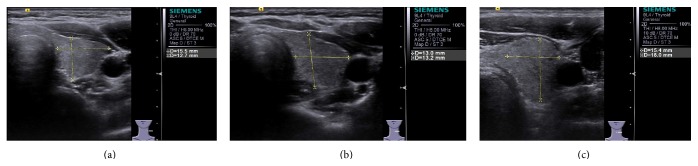
Two-dimensional ultrasound images of (a) normal thyroid, (b) GD thyroid, and (c) HT thyroid.

A wide spectrum of diffuse diseases can affect the thyroid gland, such as Graves' disease (GD), Hashimoto's thyroiditis (HT), subacute lymphocytic thyroiditis, and De Quervain thyroiditis [1]. The most common category of diffuse thyroid disease (DTD) is autoimmune thyroid diseases (AITD), including HT and GD [2]. Ordinarily, a clinical diagnosis of DTD is made based on an algorithm of presenting symptoms, laboratory analysis of thyroid function, immunology, and occasionally imaging features [3]. As the most commonly used imaging technique to screen DTD, ultrasound (US) could detect a subset of patients before they come to clinical attention and had roles in excluding focal thyroid disease and assessing the size of the thyroid [1]. However, conventional ultrasound makes the diagnosis of DTD mainly based on the morphology changes, the level of echogenicity, and vascularity of the thyroid. A certain diagnosis could be made only if all the typical image features emerged. Owing to the underlying similarities in the image features, it is sometimes difficult to differentiate between normal and DTD thyroid (Figure 1), and the accuracy of such diagnosis is largely dependent on the experience of the operator and the adjustment of machine. Fortunately, an emerging ultrasound technique, named acoustic radiation force impulse (ARFI) imaging, might be helpful to cover this shortage by offering the information of tissue stiffness. It contains two modes, virtual touch tissue imaging (VTI) and virtual tissue quantification (VTQ). The former could provide the information of tissue elasticity qualitatively, while the latter could give an objective measurement quantitatively [4, 5]. During ARFI examination, short-duration acoustic pulses are utilized to excite tissue in the region of interest (ROI) mechanically. Then, shear waves are produced and spread away from the ROI perpendicularly to the acoustic push pulse and generated localized, micron-scale displacements in the tissue [6, 7]. The displacements result in shear wave propagation away from the excited region and are tracked by ultrasound correlated methods. By measuring the time to peak displacement at each lateral location, the shear wave velocity (SWV) within the tissue can be calculated (expressed in m/s) [5]. Generally, stiffer tissue has higher SWV [6].

Recently, more and more studies have focused on the application of VTQ. It has been confirmed that VTQ had the potential to differentiate between benign and malignant focal lesions in liver, breast, kidney, and thyroid [8–16]. As we know, only a few researchers have published their preliminary results of the application of VTQ in diffuse diseases, even fewer in thyroid [6, 17–19]. So our present study aims to evaluate the application of VTQ in the diagnosis of diffuse thyroid diseases.

## 2. Materials and Methods

### 2.1. Study Population

This study was approved by the institutional review board of our hospital. All the subjects signed informed consent (for the patients under 18 years old, the informed consents were signed by their parents).

**Figure 2:**
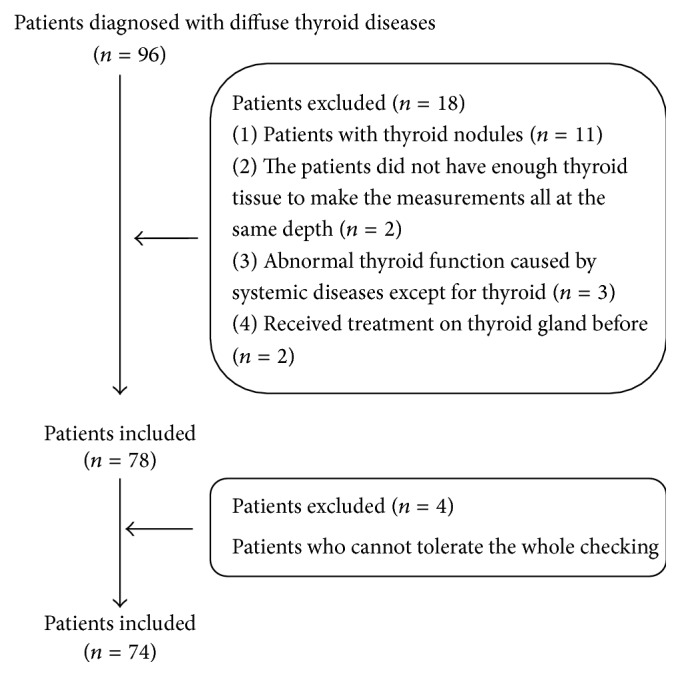
The patients' selection flowsheet.

Ninety-six consecutive patients were diagnosed with DTD by clinical physicians from May 2012 to May 2013 in our hospital. The enrollment criteria were as follows: (1) the patients had enough thyroid tissue to make the measurements all at the same depth; (2) the patients had no history of thyroid operation or drug treatment; (3) all the patients had thyroid serological testing results. The exclusion criteria were as follows: (1) the patients with thyroid nodule; (2) abnormal thyroid function caused by systemic diseases except thyroid, such as pituitary disease; (3) the patients who could not tolerate examination, for example, because of difficulties in breathing or suffering from a phobia. All the patients were diagnosed according to the criteria as follows: GD was diagnosed based on the low titers of thyroid stimulating hormone (TSH), high titers of free thyroxine (FT4), free triiodothyronine (FT3), and anti-TSH receptor antibodies in serum. The diagnosis of HT was based on high titers of anti-thyroid antibodies (thyroglobulin antibody and/or thyroglobulin antibody) in serum, normal (or low) thyroid function [19]. Finally, 74 patients (patients group, 16 men and 58 women) were enrolled in this study, including 30 cases of GD (GD group), age ranging from 19 to 55 yrs (mean 35.0 ± 13.3 yrs), and 44 cases of HT (HT group), age ranging from 12 to 67 yrs (mean 44.2 ± 14.3 yrs). The patient selection flowsheet was present in Figure 2. Additionally, 30 healthy subjects were involved in the study as control (healthy group), including 7 men and 23 women, age ranging from 23 to 64 yrs (mean 32.7 ± 14.9 yrs). The enrollment criteria were as follows: (1) no lesion on thyroid gland and (2) normal thyroid function. Those with cardiac diseases, autoimmune diseases, or blood system diseases were excluded.

### 2.2. Laboratory Tests

Thyroid serological indexes including FT3, FT4, TSH, thyroglobulin antibody (TGAb), and thyroid peroxidase antibody (TPOAb) were tested by chemiluminescence particles immune method before ultrasound examination. The interval between laboratory tests and ultrasound examination was less than 1 week.

### 2.3. Conventional US and Elastography Imaging

During ultrasound examination, the subjects kept slow and even respiration, with neck fully exposed and head leaned backward. The probe was placed tenderly on the cervical skin with light pressure. All the subjects were examined by the same operator with six years of ultrasound operation experience. The following conventional US features were recorded: the size and morphology of thyroid, the echotexture of thyroid parenchyma, and the internal blood supply. As DTD was considered as a kind of diffuse disease, the bilateral pathologic changes of thyroid were deemed equal. Thus, in VTQ measurements, we chose the left lobe instead of both lobes. The sample frame of ROI was 0.5 cm × 0.6 cm in size. The selection criteria of ROI were as follows: (1) the sample frame was, respectively, placed on upper, middle, and lower portions of the thyroid parenchyma on the largest longitudinal section, with a depth of 1.5 cm; (2) the envelope, calcification, and internal blood vessels of the thyroid were avoided. VTQ measurements were performed repeatedly for 7 times in each region, and the results were expressed by SWV (m/s). The highest and the lowest value were eliminated and the remaining 5 values were used for analysis. The range of SWV was 0–9 m/s, by the manufacturer's algorithm. Value beyond this range was displayed as “*X.XX* m/s,” which meant the tissue was extremely hard or soft. After excluding the possible relevant factors such as the operator's inappropriate operation or the patient's respiration, the value of “*X.XX* m/s” was allocated to be 0 m/s or 9 m/s, with 0 m/s corresponding cystic portion and 9 m/s corresponding solid portion [5].

### 2.4. Statistical Analysis

The statistical analyses were performed using SPSS 16.0 software package (SPSS Inc., Chicago, IL, USA). All measurements were expressed as the mean value ± standard deviation (SD).

The differences of SWV between the healthy group and DTD group were compared by the single factor analysis of variance; *P* < 0.05 was considered to be statistically significant. The diagnostic performance of VTQ in differentiating between healthy and DTD thyroid was assessed by receiver operating characteristic curve (ROC). In this curve, “1 − specificity” was displayed on the horizontal axis, and “sensitivity” was displayed on the *y*-axis. The points which composed the curve did not represent the distribution of SWV, but each cutoff value was calculated by statistical software (SPSS) automatically. When comparing different diagnostic methods, the larger the area under the curve (AUC), the better the diagnostic efficacy. For a certain diagnostic method, the cutoff value was chosen by “Youden index” (formula: specificity + sensitivity − 1). The best cutoff value had the largest Youden index. The correlation between SWV and serological testing indexes was analyzed by linear regression.

## 3. Results

The baseline characteristics of the subjects were presented in Table 1. There was no significant difference between healthy group and DTD group in age or sex.

**Figure 3:**
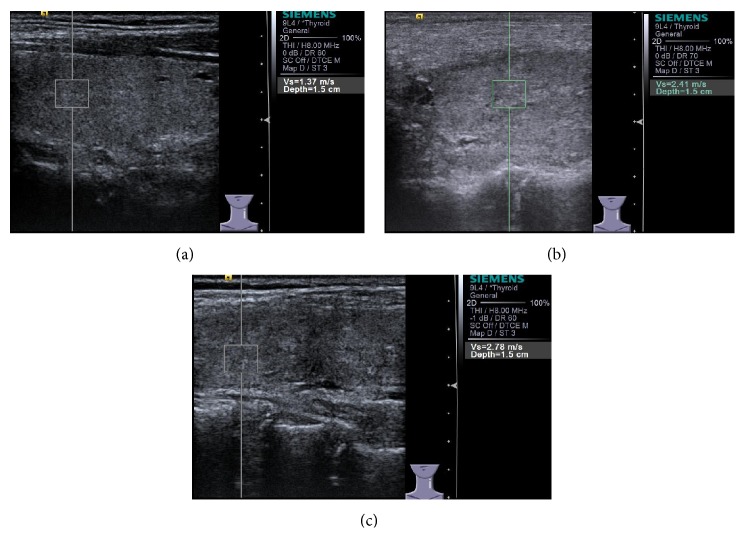
The VTQ of thyroid tissue. (a) A 30-year-old female with normal thyroid, SWV = 1.37 m/s and depth = 1.5 cm; (b) a 42-year-old female GD patient, SWV = 2.41 m/s and depth = 1.5 cm; (c) a 35-year-old female HT patient, SWV = 2.78 m/s and depth = 1.5 cm.

The thyroid SWVs of DTD patients were higher than those of the healthy (2.56 ± 1.33 m/s versus 1.74 ± 0.16 m/s, *P* = 0.011). The SWVs of the upper, middle, and lower part of the thyroid tissue in patients with GD and HT were all higher than those of the healthy (*P* < 0.05) (Figure 3). However, there were no significant differences between GD and HT groups in SWV (2.14 ± 0.31 m/s versus 2.72 ± 1.52 m/s, *P* = 0.179). No matter in which group, no variance was detected between different thyroid parts in SWV (Table 2).

**Figure 4:**
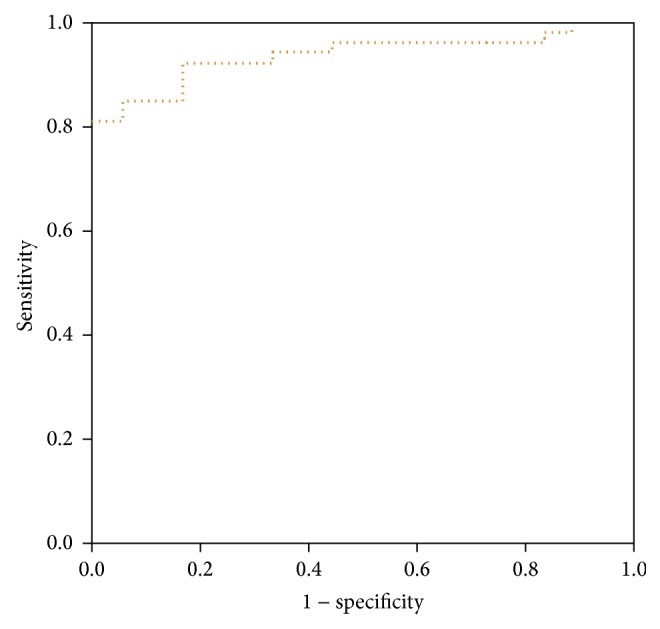
ROC curve for SWV in differentiating between normal and DTD thyroid.

According to the analysis by ROC, with a cutoff SWV value of 2.02 m/s to distinguish DTD and healthy thyroid, the area under the curve was 0.938 (Figure 4) and the sensitivity and specificity were 81.12% and 100.00%, respectively.

**Figure 5:**
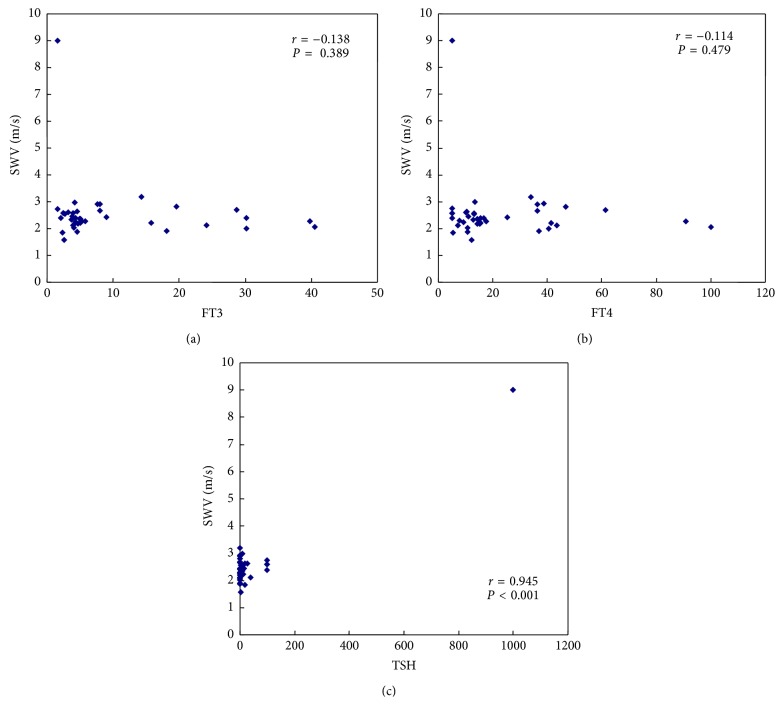
The linear correlation between SWV and thyroid serological indexes in DTD patients. (a) FT3 (*γ* = −0.138, *P* = 0.389); (b) FT4 (*γ* = −0.114, *P* = 0.479); (c) TSH (*γ* = 0.945, *P* < 0.001).

**Figure 6:**
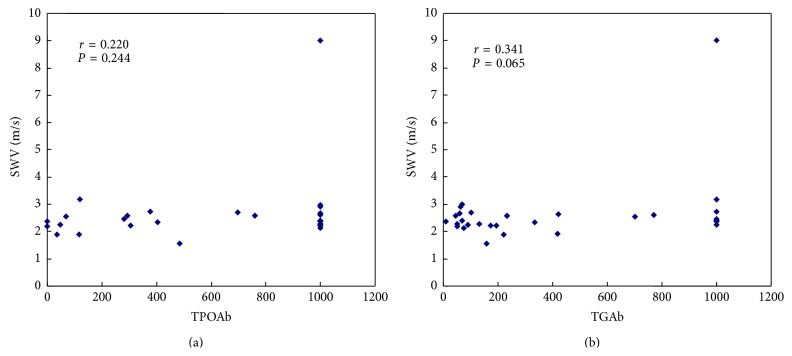
The linear correlation between SWV and autoimmune antibodies in HT patients. (a) TPOAb (*γ* = 0.220, *P* = 0.244); (b) TGAb (*γ* = 0.341, *P* = 0.065).

According to the analysis by linear regression, we found that, in DTD patients, the SWV values had positive linear correlation with TSH (*γ* = 0.945, *P* < 0.001), but not with FT3 or FT4 (Figure 5). Moreover, the SWV values had no correlation with TPOAb or TGAb in HT patients (Figure 6).

## 4. Discussion

Nowadays, many studies have focused on the feasibility of using VTQ to differentiate between malignant and benign thyroid nodules [5, 15, 16], but very few were concerned with the DTD. Accordingly, we set out to perform VTQ examination to evaluate the thyroid tissue elasticity in DTD patients. We found that, compared with healthy people, DTD patients generally had stiffer thyroid tissue.

Stiffness or elasticity is one of the important physical parameters of biological tissue, which is largely dependent on the construction of cells and molecules in the tissue. Thus, stiffness usually changes with pathologic status. As the patients in our study were all not treated, the stiffness of thyroid might reflect the tissue status objectively. Normal thyroid gland is characterized by even thyroid follicles and abundant capillaries which are evenly distributed between the follicles. While for GD the pathological histology is characterized by diffuse hypertrophy and hyperplasia of follicular cells with colloid depletion and lymphoid infiltration [1], for HT the pathological histology is characterized by lymphoplasmacytic aggregates with germinal centers, atrophic thyroid follicles, oxyphilic change of the epithelial cells, and variable fibrosis [20]. Moreover, collagen fibril is highly elastic [21], which means that collagen fibril needs much force to make deformation and enables substantial energy dissipation during deformation. In other words, under the same the external force, thyroid tissue with more collagen fibril generated less deformation. These pathological changes might make the thyroid tissue stiffer both in GD and HT. Correspondingly, we found that the thyroid SWV in DTD patients was higher than that of the healthy. In consideration of the fact that conventional US examination is largely dependent on the experience of operator, such objective indexes might make the sonographer more confident in making a DTD diagnosis. Nevertheless, our findings showed that the thyroid tissue in HT and GD had similar elasticity. That is to say, we could not differentiate between various DTD subtypes merely based on stiffness. When introduced to clinical application, comprehensive information was still essential.

Although the pathologic changes in DTD thyroid tissue were usually deemed even, we still analyzed the influence of sampling. Firstly, in our preliminary study we performed VTQ examination on each side of the thyroid, respectively, to compare their stiffness, and found that there was no significant difference, no matter in healthy group or diffuse thyroid disease (DTD) group (data not shown). This result was consistent with another study [19]. Thus, to improve the efficiency in data collection and calculation, we uniformly chose the left lobe to perform VTQ in our present study. Additionally, we also performed VTQ at different positions in thyroid tissue and found that the measurement of a random position was equal to that of the whole. These results were in accordance with the uniform pathologic changes of DTD and might simplify the measurement process during clinical application. What is more, as VTQ measurement was sometimes influenced by the patient's respiration or the probe's movement, it was necessary to be performed repeatedly. In Sporea et al.'s research [19], VTQ measurements were repeated for 5 or 10 times to determine the proper times of repetition. They found that there was no significant difference in data between 5 times and 10 times. That is to say, 5 times of repetition was adequate. Concerning the reliability of data, we thought that the highest and the lowest value would better be eliminated to avoid the occurrence of abnormal value. For the above reasons, we performed 7 times of repetition and eliminated the highest and the lowest value. The mean of the rest 5 values was used for analysis. The same method has also been mentioned in Zhang et al.'s research [5].

According to our study, a cutoff SWV value of 2.02 m/s had the best capacity to diagnose DTD, while in Sporea et al.'s [19] research, the optimal cutoff value was 2.36 m/s. What is more, their thyroid SWVs in DTD patients and healthy people were also both higher than those of ours. Even so, the cutoff value in both studies had similar and satisfactory diagnostic capacity. We speculated that the diversity between the two studies might be explained by the differences in age and men/women distribution of the study cohort or the different operators, parameter settings, and measuring methods. As thyroid is a parenchymal organ containing plenty of vessels, there might be discrepancy in cell distribution on different sections. What is more, pulling strength acting on thyroid from each direction might be unbalanced, especially on different body position. Thyroid is anatomically fixed by the surrounding tissue, such as pretracheal fascia, lateral ligament, and suspensory ligament, which pull thyroid from different directions. During VTQ examination, acoustic push is applied to thyroid, which might simultaneously induce the surrounding tissue to react. If we perform VTQ on different sections, the pulling strength generated by the surrounding tissue might also be different, leading to a slight discrepancy in tissue deformation and SWV measurements. This phenomenon has not been reported in other studies and thus needs more clinical tests or even mechanical experiments to make it validated. Thus, specifying the section of measurements is necessary.

In our study, the thyroid SWV in DTD patients had positive linear correlation with TSH titer, but not with FT3 or FT4. As TSH is the most sensitive indicator of thyroid function and disease progression [22, 23], SWV value might have potential for assessing the thyroid function noninvasively and also reflect the progression of disease and therapeutic effect. While concerning FT3 and FT4, as all the patients in our study were diagnosed for the first time, most of them might be at the early stage of disease, and their FT3 and FT4 in serum might not change timely and sensitively. Additionally, we found that SWV had no correlation with TGAb or TPOAb titer in HT patients. Although TGAb and TPOAb have high specificity on HT diagnosis [24, 25], the development of HT is a complex pathological process. Besides TGAb and TPOAb, many other kinds of autoimmune antibodies are also involved, such as thyroid stimulating antibody, thyrotropin receptor antibody, and thyroid inhibiting antibody. The thyroid status in HT might be influenced by the coaction of various antibodies; thus, the titer of TGAb and TPOAb alone might not reflect the thyroid pathologic status, not to mention the thyroid stiffness.

We must confess the limitations in our study. Firstly, only the GD and HT patients were involved. Other types of DTD, such as subacute lymphocytic thyroiditis and De Quervain thyroiditis, need to be investigated further. Secondly, as a preliminary study, our sample size was relatively small. Study on large sample and the comparison between different races or nations are required. Thirdly, whether there is dynamic change in thyroid stiffness during treatment also needs to be investigated.

## 5. Conclusion

In conclusion, VTQ could be used to assess the thyroid tissue stiffness quantitatively and might have a potential in assessing the thyroid function in DTD patients. As an objective index, SWV might be used complementarily with traditional ultrasound to make the sonographer more confident in making a DTD diagnosis.

## Figures and Tables

**Table 1:** Baseline characteristics of DTD and healthy groups.

Group	*N*	Age (year, $\overset{-}{x}\pm s$)	Sex (*n*, male/female)
Healthy	30	39.56 ± 14.90	7/23
DTD	74	41.77 ± 14.37	16/58
*P*		0.577	0.851

**Table 2:** The SWV of different thyroid locations in healthy and DTD groups (m/s, $\overset{-}{x}\pm s$).

Group	*N*	Upper	Middle	Lower	Whole
Healthy	30	1.79 ± 0.18	1.71 ± 0.19	1.74 ± 0.23	1.74 ± 0.16
DTD	74	2.54 ± 1.35^*∗*^	2.64 ± 1.35^*∗*^	2.50 ± 1.35^*∗*^	2.56 ± 1.33^*∗*^
GD	30	2.16 ± 0.40^†^	2.23 ± 0.31^†^	2.05 ± 0.39^†^	2.14 ± 0.31^†^
HT	44	2.69 ± 1.54^‡^	2.79 ± 1.54^‡^	2.67 ± 1.53^‡^	2.72 ± 1.52^‡^

^*∗*^ *P* < 0.05, DTD group compared with the healthy group; ^†^ *P* < 0.05, GD group compared with the healthy group; ^‡^ *P* < 0.05, HT group compared with the healthy group.
